# Can ‘eugenics’ be defended?

**DOI:** 10.1007/s40592-021-00129-1

**Published:** 2021-05-25

**Authors:** Walter Veit, Jonathan Anomaly, Nicholas Agar, Peter Singer, Diana S. Fleischman, Francesca Minerva

**Affiliations:** 1 University of Sydney, Sydney, Australia; 2 University of Pennsylvania, Philadelphia, USA; 3 Victoria University of Wellington, Wellington, New Zealand; 4 Princeton University, Princeton, USA; 5 University of Portsmouth, Portsmouth, UK; 6 University of Warwick, Coventry, UK; 7 University of Ghent, Ghent, Belgium

**Keywords:** Ethics, Eugenics, Enhancement, Human enhancement, Embryo selection, Gene editing, CRISPR

## Abstract

In recent years, bioethical discourse around the topic of ‘genetic enhancement’ has become increasingly politicized. We fear there is too much focus on the semantic question of whether we should call particular practices and emerging bio-technologies such as CRISPR ‘eugenics’, rather than the more important question of how we should view them from the perspective of ethics and policy. Here, we address the question of whether ‘eugenics’ can be defended and how proponents and critics of enhancement should engage with each other.

## Introduction

Recently, the *Monash Bioethics Review* published an article titled ‘Defending eugenics: From cryptic choice to conscious selection’ (Anomaly 2018), a paper that caused a stir among some in the academic community. Petitions condemning the paper were initiated and activists on social media denounced Anomaly’s paper and called for him to be fired. Robert Wilson (2019), in turn, offered a reply with the title ‘Eugenics Undefended’ in which he criticized almost every premise in Anomaly’s paper.

Here, we are not primarily concerned with the respective arguments of Anomaly and Wilson, but with the higher-level question of whether ‘eugenics’ can be defended at all. Much of the confusion in the debate, we fear, rests on the merely semantic question of whether we should call—or in this case perhaps it would be better to say *brand*—the use of emerging bio-technologies like embryo selection and CRISPR as ‘eugenics’ or ‘genetic enhancement’ rather than the more philosophically important question of how we should view them from the perspective of ethics and policy.

## The term ‘Eugenics’

The term ‘eugenics’ (which means ‘good birth’) was coined by Francis Galton in 1883 to capture the idea that we should use insights from the new science of heredity to improve the welfare of future people (Levine 2017). But as Galton understood the term, eugenics involved both the study of heredity, and the use of this knowledge to by parents to shape their reproductive choices. It is more common now to sharply distinguish the study of *genetics* (a term that wasn’t coined until 1905) from *eugenics*. For example, in their recent book *The Ethics of the New Eugenics* MacKellar and Bechtel define eugenics as involving ‘strategies or decisions aimed at affecting, in a manner which is considered to be positive, the genetic heritage of a child, a community, or humanity in general’ (2016, p. 3). If we use this definition, many contemporary bioethicists support eugenics (e.g. Savulescu 2001; Brock 2005; Buchanan and Powell 2011; Gyngell and Selgelid 2016).

But there is an obvious reason authors often shy away from using the term ‘eugenics’. This is the association with forced sterilization programs in the US and Nazi Germany, as well as the Nazi program of euthanizing disabled people, and the mass murder and attempted genocide of Jews and Roma during WW2. Eugenics has always had advocates who *rejected* a role for the state in guiding procreative choices, advocates who thought the state should play a *limited* role in influencing parental choice by providing information to prospective parents or subsidies for genetic interventions, and advocates who thought the state should play a *significant* role, including the use of extensive compulsion. While contemporary bioethicists disagree about whether the state should play a role in helping parents discharge their procreative obligations, none think the state should engage in the mass sterilization or murder of their own citizens. In other words, the rejection of Nazi-style eugenics programs is unanimous. Nevertheless, ‘eugenics’ has increasingly become associated in the public mind with its worst abuses.

To call a person a ‘eugenicist’ or deem a practice ‘eugenics’ is often accepted as a substitute for an argument. However, all human societies engage in a variety of practices that are both widely accepted and plainly eugenic. In the West, most pregnant women test for disorders such as Down syndrome, Huntington’s disease, and cystic fibrosis. Many people choose to terminate pregnancies that are likely to result in a genetic disorder or disability. Incest is forbidden in most cultures and cousin marriage is illegal in many nations for transparently eugenic reasons: the children that result are more likely to suffer from a disorder or disability. Perhaps the most straightforwardly eugenic policy is the provision of genetic counselling among at-risk ethnic groups to prevent the birth of, for example, children with Tay-Sachs, sickle cell disease and thalassemia.

The important conclusion is this: *everyone who considers pre-natal testing justifiable, or who thinks women should be free to weigh genetic information in the selection of a spouse or a sperm donor is a eugenicist*.

The difference between those embracing and those criticizing the term is merely expressed in where we draw a boundary between the kinds of eugenic practices we allow. Indeed, it is hard to find people who don’t endorse *any* form of eugenics for the same reason it is hard to find people who don’t think mothers should be careful about what they consume and how they behave when they are pregnant. Nearly everyone agrees that pregnant women should avoid foods containing mercury during pregnancy (since mercury impairs brain development), and there are public health campaigns to discourage women from smoking, drinking alcohol to excess or getting x-rays because these may cause cognitive or physical disability in their children. The source of the disability, environmental versus genetic, is the only distinction here. Avoiding excessive alcohol while pregnant is morally analogous to selecting among a set of embryos in a way that minimizes the likelihood that a future child will have serious cognitive disabilities. Unless one comes to endorse the claim that all of the above practices are wrong, it is hard not to implicitly endorse *some kind of eugenics*.

Many authors think that whatever words we use, eugenics in some form is inevitable given recent advances in gene editing and embryo selection, and that changing the word doesn’t change the underlying debate (Agar 2019; Buchanan et al 2000; MacKellar and Bechtel 2016; Selgelid 2014). For example, Philip Kitcher has argued that ‘Once we have left the garden of genetic innocence, some form of eugenics is inescapable’ (p. 174). This is because, Kitcher thinks, the choice to use *or not use* genetic screening, contraception, or abortion predictably influences what kinds of people are born, and what kinds of traits they will have. As Kitcher understands the term (following Galton), eugenics is ‘a mixture of the study of heredity and some doctrines about the value of human lives’ (1997, p. 191). He suggests that even if a parent or policy is not attempting to alter the human gene pool, insofar as policies and parental choices predictably affect the genetic endowments of future people, and thus the composition of future populations, they constitute a form of eugenics. Likewise, the historian of eugenics, Daniel Kevles, argues that if policies that subsidize genetic counselling and contraception affect the gene pool, they are *eugenic* (or *dysgenic*) policies, even if this is not their intent (1985, p. 258). Although scholars tend to define their terms carefully, it is increasingly common in popular discourse to use ‘eugenics’ to designate only interventions that involve unjust coercion. As we’ve argued, we think this is misguided.

But if the term ‘eugenics’ is so incendiary, why use it at all? Why not use the euphemism ‘genetic enhancement’? Is the point to cause controversy and draw attention? Not necessarily. It’s important that the debate about eugenics continue unconstrained by requirements such as those that Wilson (2019) would impose. The silencing of reasoned defenses of eugenics threatens a dangerous neglect of the risk of repeating past errors by disassociating them from their historical misuse (Agar 2019; Anomaly 2021). Nevertheless, precisely because of the historical atrocities committed in the name of eugenics, some philosophers advocate using ‘genetic enhancement’ in its place (Wilkinson 2008; Camporesi 2014; Cavaliere 2018). Terms like ‘gene therapy’ and ‘genetic enhancement’ lack the discomforting associations of ‘eugenics’. In our opinion, this does not make genetic interventions any more or less dangerous. It just changes the words we use to describe them. The important point is not what words we use but instead the moral distinctions we make between different kinds of interventions. After all, Hitler imposed a grotesque involuntary 'euthanasia' program, but many people now think *voluntary* euthanasia is justifiable. Nicholas Agar (2019, p. 10) distinguishes between interventions that are morally wrong and interventions that are morally problematic: ‘All instances of an intervention properly identified as essentially morally wrong are morally wrong. However, a morally problematic intervention is problematic precisely because it comprises both morally bad and morally good interventions’. Slavery is essentially morally wrong—there are no cases of ‘morally good’ slavery. Eugenics is morally problematic in that it comprises good and bad practices. It is likely to be essentially so. There is unlikely to be a future in which people making choices about what kinds of people will exist run no risk of the errors of authoritarian eugenics. The use of the term ‘eugenics’ breeds caution, but it should not be misused as the replacement of a moral argument.

## How to defend and criticize ‘eugenics’

Wilson (2019) demands that proponents of genetic enhancement such as Peter Singer (2001, 2003), Jonathan Glover (2006), Nicholas Agar (1998, 2004, 2019), Julian Savulescu (2001, 2009), John Harris (1992, 2007), Walter Veit (2018a,b,c), and Jonathan Anomaly (2018, 2020) should pay attention to ‘the actual history of eugenics and the considerable scholarship on it’, which should ultimately raise the standards of credibility that ‘any publishable work defending eugenics should meet’ (p. 68). However, in almost all essays that advocate some version of eugenics, the authors have specified which version they endorse, and which principles and practices of eugenics are morally unacceptable. It is possible that proponents of genetic enhancement have failed to adequately engage with the entirety of this literature. But what is it to ‘adequately’ engage with the work of others? Most of the proponents of genetic enhancement have explicitly acknowledged the darkest chapters in the history of ‘eugenics’ and emphasized that we should learn from its tainted history. As a result, philosophers have distinguished between positive vs negative eugenics, liberal vs coercive eugenics, and individualist vs collectivist eugenics, among other distinctions.

We should acknowledge that implications about better or worse lives are not limited to hereditary choices. One of us has type 1 diabetes and is sensitive to the eradicationist ambitions of public health campaigns targeting the condition. A world in which there are no new type 1 diabetics is one in which he may be deprived of the good of fellowship with others who share his condition. There will be reduced incentives to find better treatments for diabetes. But we nevertheless recognize the value in efforts to prevent the disease. Some genetic disorders, after all, are already rare – with little investment being made into research to cure them. The same can be said of public health interventions. Public health campaigns can be morally problematic in the sense described above. Some anti-obesity campaigns can inadvertently stigmatize vulnerable young people. Every principle or policy has unintended consequences, we can only seek to implement those whose benefits outweigh their costs.

We agree with Robert Wilson and other critics of eugenics that it’s imperative to define our terms clearly, and to specify the relevant values at stake. Once that is done, however, the arguments should be over substantive claims rather than labels. To illustrate this point, we can look at another term that has a strong mental association with the Nazis and often comes up in this debate: ‘genocide’.

If the use of cochlear implants means that there are fewer Deaf people, is this ‘genocide’? Does our acceptance of prenatal diagnosis and selective abortion mean that we are ‘drifting toward a eugenic resurgence that differs only superficially from earlier patterns’. If the use of the term ‘genocide’ is intended to suggest a comparison with the Holocaust, or Rwanda, it overlooks the crucial fact that cochlear implants do not have victims. On balance, it seems that they benefit the people who have them; if this judgment is contestable, it is at least not clear that they are worse off for having the implant. Imagine a minority ethnic group in which all the parents reach separate decisions that their children will be better off if they marry a member of the majority group, and hence urge them to do so. Is this encouraging ‘genocide’? If so, it is genocide of such a harmless form that the term should be divorced from all its usual moral associations.

– Peter Singer (2003)

The notion that there could be a morally unproblematic form of genocide will appear to many as an even more outrageous suggestion than to claim that there could be a morally unproblematic form of eugenics. In a recent paper by Yeh et al. (2020), researchers found a potential pathway towards a cure of some forms of deafness by using the gene-editing tool called CRISPR. By replacing cochlear implants with an even earlier genetic intervention the two epithets of genocide and eugenics merge. Instead of asking whether we should use such technologies, much of the debate seems to have devolved into a discussion about semantics on whether such approaches to disabilities and diseases should be considered ‘eugenics’ or ‘genocide’ thus reducing a complex moral problem into an apparently easy one by merely having to determine whether these technologies and practices fit into these supposedly evil categories. This can be similarly seen in an op-ed piece by Sarah Katz (2020) in *Discover Magazine* on the research of Yeh et al (2020) that accuses them of ‘audism’—which she defines as the ‘belief that people with the ability to hear or to emulate those who can hear are superior’. Here, we need to be careful to distinguish a substantive (and largely empirical) claim from a semantic one that is intended to have normative implications. Is the very act of asking the question which conditions make a life go better or worse (for the person) automatically devaluing the lives of those with diseases and disabilities? Katz appears careful to recognize a distinction between the two. Others in the debate have asserted that one cannot disentangle them. However, it is this mistaken view within the debate, that Singer (2003) tries to appeal against: the mere notion that we can make moral progress by deciding which terms to use.^1^ The question should rather be: If we had access to such technologies, should we use them?

## Conclusion

Proponents of genetic enhancement, if they endorse the label of eugenics, do so precisely because their positions are inevitably going to be labelled as ‘eugenics’ by critics who want to shut down debate quickly. Disability theorists who view their scholarship as a sort of activism for the rights and concerns of the disabled (following Wilson 2019) will continue to demand more engagement and citation of arguments critical of genetic enhancement and eugenics. Regardless of the term we use, the academic debate around genetic enhancement is polarized now, and there is a danger that it will only become more so as these technologies come to the fore. There is a legitimate worry that the requirement for ‘sufficient engagement’ will only be met when proponents of genetic enhancement come to abandon their views and adopt the positions of their opponents.

But just as enhancement isn’t a unified category that we can simply judge as morally good or bad (Veit et al. 2020), so too with genetic enhancement or eugenics (Anomaly, Gyngell, and Savulescu 2020). If our goal is to find the best answers to the complex questions raised by new biomedical technologies, it won’t do to operate in the echo chambers of our respective academic niches. We’ll have to stop thinking in purely partisan terms, where anyone who doesn’t agree with our conclusions is ridiculed and publicly condemned. But how can we fight this polarization in academia?

When a debate is socially consequential, we should engage with different points of view and treat scholars we disagree with charitably. This applies to both defenders and critics of enhancement. Critics of genetic enhancement, whether they are philosophers or disability scholars, should attempt to see the arguments of enhancement proponents not as Nazi propaganda in disguise, but rather as honest attempts to defend the use of these technologies to improve well-being and autonomy. Mark Kuczewski (2001) has argued that bioethicists need to engage more with disabled people and enter into a dialogue *with* rather than about them. Maybe so. But reasonable people will disagree about which traits promote human flourishing even after they have thought about the issues surrounding disability and engaged with different kinds of people. As Peter Singer has argued:

Individual bioethicists who come across something that they regard as wrong may choose to dedicate themselves to advocacy for the cause of those who they see as wronged, but if they become mere partisans, dismissing without adequate consideration the views of others who are not advocates for the same group, they risk becoming propagandists rather than scholars.

– Peter Singer (2001, p. 55).

To conclude: academic polarization is just as real as political polarization and it can undermine careful reflection when we are faced with complex ethical problems. To find solutions to these problems, we need to listen to each other and take literature in other fields seriously. Turning this issue into a semantic debate won’t lead us toward a solution, but rather away from it.

## Footnotes

1 Veit and Browning (2020) make similar arguments in their distinction between two kinds of conceptual engineering – here the conflict is between a term accurately representing the world and a term being used to promote moral goals.
